# Causes of Death among Women Aged 10–50 Years in Bangladesh, 1996–1997

**Published:** 2007-09

**Authors:** Hussain R. Yusuf, Halida H. Akhter, Mahbub Elahi Chowdhury, Roger W. Rochat

**Affiliations:** 1 Department of Global Health, Rollins School of Public Health, Emory University, Atlanta, Georgia, USA; 2 Health Promotion Limited, House 310, Road 3, Baitul Aman Housing Society, Shyamoli, Dhaka 1207, Bangladesh; 3 Bangladesh Institute of Research for Promotion of Essential and Reproductive Health and Technologies, House 105, Road 9/A (New), Dhanmondi Residential Area, Dhaka 1209, Bangladesh

**Keywords:** Causes of death, Maternal mortality, Pregnancy, Pregnancy complications, Bangladesh

## Abstract

Limited information is available at the national and district levels on causes of death among women of reproductive age in Bangladesh. During 1996–1997, health-service functionaries in facilities providing obstetric and maternal and child-heath services were interviewed on their knowledge of deaths of women aged 10–50 years in the past 12 months. In addition, case reports were abstracted from medical records in facilities with in-patient services. The study covered 4,751 health facilities in Bangladesh. Of 28,998 deaths reported, 13,502 (46.6%) occurred due to medical causes, 8,562 (29.5%) due to pregnancy-related causes, 6,168 (21.3%) due to injuries, and 425 (1.5%) and 259 (0.9%) due to injuries and medical causes during pregnancy respectively. Cardiac problems (11.7%), infectious diseases (11.3%), and system disorders (9.1%) were the major medical causes of deaths. Pregnancy-associated causes included direct maternal deaths (20.1%), abortion (5.1%), and indirect maternal deaths (4.3%). The highest proportion of deaths among women aged 10–19 years was due to injuries (39.3%) with suicides accounting for 21.7%. The largest proportion of direct obstetric deaths occurred among women aged 20–29 years (30.5%). At least one quarter (24.3%) of women (n=28,998) did not receive any treatment prior to death, and 47.8% received treatment either from a registered physician or in a facility. More focus is needed on all causes of deaths among women of reproductive age in Bangladesh.

## INTRODUCTION

In developing countries, such as Bangladesh, deaths among women, especially those from pregnancy-related causes are commonly underreported (1). Several studies have reported that pregnancy-related complications are among the leading causes of death among women of childbearing age in Bangladesh (2-6). Limited research has examined other causes of death and disability. A study in the rural subdistrict of Matlab used verbal autopsies in the homes of the deceased to identify the causes of death among women aged 15–44 years during 1976–1985. Most deaths occurred either due to infectious diseases (32%) or due to direct obstetric complications (30%). Intentional and unintentional injuries and acute and chronic non-infectious diseases, including cancer and cardiovascular diseases, caused 12% and 7% of deaths respectively. The other deaths were from unspecified conditions (7).

Epidemiological assessment of causes of death among women of reproductive age in Bangladesh is needed for increased awareness of health problems in this population group, allocating public-health resources and appropriately developing strategies for prevention. Such information is also needed for monitoring and evaluating ongoing health-promotion programmes. To assess the causes of death among female adolescents and women, we conducted a country-wide study to identify deaths due to any cause among women aged 10–50 years. Three papers were earlier published from this study focusing on deaths associated with obstetric causes, tetanus-related deaths, and injury-related deaths (8-10).

## MATERIALS AND METHODS

We used a case-finding approach to identify cases of mortality among women aged 10–50 years in Bangladesh. During October 1996–March 1997, health-service functionaries in facilities throughout the country providing obstetric, maternal and child health were interviewed on their knowledge of deaths among women in this age-group in the past 12 months. In addition, case reports of deaths were abstracted from medical records in facilities that have inpatient services.

Our study, covering 4,751 facilities in the country, included all the 13 government medical college hospitals, five infectious disease hospitals, and 96 maternal and child welfare centres. Most (58 of 64) government district hospitals, Upazila (subdistrict) Health Complexes (453 of 469), Union Health and Family Welfare Centres (3,113 of 4,451), and Union Council clinics (909) were covered. In addition, the study included eight non-government organization-run healthcare facilities, eight private-sector clinics or hospitals, and 88 other facilities providing healthcare to women. Interviews were conducted in facilities located in 60 of the 64 districts in Bangladesh. Facilities in four districts (Rangamati, Khagrachhari, Banderban, and Sunamganj) could not be reached because of floods and other conditions.

### Interview with healthcare providers

At each facility, one person was interviewed as the principal informant of cases of deaths among women in their service area, and up to three persons were interviewed as secondary informants to acquire additional information regarding the reported cases. We interviewed 201 physicians, 754 nurses/midwives, 3,555 family welfare visitors (FWVs) (trained health workers stationed at Union Health and Family Welfare Centres), 497 medical assistants, and 440 other healthcare providers as principal informants. We also interviewed 16,732 Family Welfare Assistants (FWAs) from an estimated 27,912 posts during the time of the study. The FWAs have formal training on maternal and child healthcare, each being responsible for about 500 households. They make home-visits for healthcare regularly in the community. The FWAs were asked to come to their respective family welfare centres for interview and were selected for interview based on their presence at the health facilities.

We asked each informant “How many women aged 10–50 years do you know of who died in the past 12 months?” For each reported death, standardized questionnaires were used for collecting information on causes and surrounding circumstances and for obtaining reproductive and sociodemographic characteristics of the woman. The interviewers were university graduates in social or biological sciences who received specific training for conducting this investigation.

### Information from medical records

Information from medical records was collected at medical college hospitals, district hospitals, infectious disease hospitals, maternal and child welfare centres and Upazila Health Complexes about women who died in the past 12 months. Information was also abstracted from death registers of individual wards or from the hospital's master register. Death records were also included from ward registers and admission forms for D &C (dilatation evaluation and curettage). Most registers had causes of deaths but did not have complete information on personal characteristics. Medical graduates retrieved hospital records using a structured form for each deceased woman.

After data collection, physician-specialists, including the principal investigator of the project, the project coordinator, and the project officer used a defined decision tree for best assessing the reported causes and circumstances surrounding each death and for attributing the most likely cause of death. We classified causes of death as per the WHO International Classification of Disease (11) into five broad categories: deaths from medical causes, maternal deaths (direct and indirect), abortion-related deaths, pregnancy-associated deaths from medical causes, and injury-related deaths. We assessed characteristics of women who died due to abortion separately from other direct obstetric deaths to examine the differences in these characteristics. We identified duplicated reports of death by conducting search for duplicated name and address of residence (e.g. village name). We assessed causes of death by determining the percentage of deaths reported due to various causes. We estimated unadjusted rates of death due to various causes by applying the reported number of deaths to an estimated population of women aged 10–49 years in 1996 (based on projections on the 1991 census and female-to-male population ratios by age-group).

## RESULTS

The study obtained case reports of 28,998 deaths among women aged 10–50 years, of which 8.4% occurred in 1995, 89.0% in 1996, 0.2% in 1997; the year was not specified for 2.4% of the case reports. An additional 174 cases were identified as duplicate reports and discarded from analysis. Overall, 6,819 (23.5%) case reports came from hospital records and 22,179 (76.5%) from interviews with facility providers and community health functionaries. Of the provider-reported deaths, the largest number was reported by FWAs (75.4%) and FWVs (16.0%). Physicians, nurses, medical assistants, and other staff reported the remaining cases.

Table 1 demonstrates the consistent pattern of reporting on broad categories of causes of deaths by different sources of information. The FWVs reported the highest percentage of direct obstetric deaths (26.7%) which was expected as the FWVs were directly involved in providing pregnancy care. The corresponding figures by the FWAs and hospital records were 17% and 25% respectively. Doctors reported the highest percentage of injury-related deaths (35.8%), which was also expected. The hospital records and FWAs were the major sources of information on deaths due to medical causes.

**Table 1:** Percentage of deaths of women aged 10–50 years obtained from various sources by cause of death

Source of information	No. of deaths	% of deaths by cause					
		Direct obstetric	Indirect obstetric	Abortion	Medical	Injury
Respondent at facility						
Doctor	201	20.4	2.0	8.0	33.8	35.8
Nurse/midwife	754	23.5	3.4	3.6	42.7	26.8
Family Welfare Visitor	3,555	26.1	5.1	8.7	30.9	29.1
Medical Assistant	497	22.5	5.2	8.5	33.0	30.8
Others	440	19.3	5.0	7.7	33.2	34.8
Respondent at community						
Family Welfare Assistant	16,732	16.7	5.0	4.6	50.8	23.0
Hospital records	6,819	24.9	2.4	4.1	50.8	17.7
Total	28,998	20.1	4.3	5.1	47.5	23.0

For hospital record-identified deaths, the deaths were evenly distributed over the 12-month period from October 1995 through September 1996. The monthly death events reported by the interviewees suggested an under-reporting of deaths occurring more than six months before the interview. For the six months immediately preceding the interview, respondents reported 13,051 deaths, or an average of 2,120, per month; for the 6–12-month period before the interview, respondents reported 7,059 deaths, or an average of 1,170, per month. Therefore, 45.9% fewer deaths were reported for the period of 6–12 months before the interview [100_∗_ (13,051–7,059) / (13,051)=45.9%].

Of the 28,998 deaths, 13,502 (46.6%) occurred due to medical causes, 8,562 (29.5%) due to pregnancy-associated causes, 6,168 (21.3%) due to injuries, and the remaining deaths occurred due to medical causes (0.9%) and injuries (1.5%) during pregnancy (Table 2). Sixty-five (0.2%) cases that were reported to be due to injuries—but where the specific cause was not reported, were classified as unspecified cause. Cardiac problems (11.7%), infectious diseases (11.3%), and system disorders (9.1%) were the major reported causes of deaths due to medical conditions. Pregnancy-related deaths among women included deaths from direct maternal causes, deaths from indirect obstetric causes, and abortion-related deaths. In the first category, eclampsia (8.4%), postpartum and antepartum bleeding (4.1%), and obstructed labour (2.8%) were the three most commonest causes of deaths. Indirect maternal deaths accounted for 4.3% of the deaths and included deaths relating to anaemia, malnutrition, cardiac problems, hepatobiliary disorders, nephrologic disorders, infective conditions, and other diseases. Approximately, 0.7% were deaths due to tetanus. Abortions contributed to 5.1% of deaths; over 95% of these deaths were due to induced abortions.

**Table 2:** Reported causes of death among women aged 10–50 years in Bangladesh, 1996–1997

Cause of death	No. of deaths (n=28,998)	% of all deaths	Deaths/100,000 women (unadjusted)∗
Pregnancy cause	8,562	29.5	24.7
Direct obstetric	5,831	20.1	16.8
Eclampsia	2,429	8.4	7.0
PPH/APH	1,195	4.1	3.4
Obstructed labour	804	2.8	2.3
Retained placenta	635	2.2	1.8
Tetanus	194	0.7	0.6
Other	574	2.0	1.7
Abortion	1,476	5.1	4.3
Induced	1,415	4.9	4.1
Spontaneous	61	0.2	0.2
Indirect obstetric	1,255	4.3	3.6
Pregnancy associated-medical cause	259	0.9	0.7
Other medical causes	13,502	46.6	38.9
Cardiac problems	3,385	11.7	9.8
Infectious diseases	3,290	11.3	9.5
System disorders	2,625	9.1	7.6
Cancer	1,527	5.3	4.4
Anaemia/malnutrition	1,374	4.7	4.0
Hepatobiliary conditions	1,301	4.5	3.8
Pregnancy-associated injury	425	1.5	1.2
Other injuries	6,186	21.3	17.8
Suicide	3,116	10.7	9.0
Accidental	1,025	3.5	3.0
Homicide	287	1.0	0.8
Unknown intent	1,758	6.1	5.1
Unspecified cause	64	0.2	0.2

∗ The unadjusted rates of death by specific causes were estimated by applying the identified number of deaths to the estimated count of women aged 10–49 years in 1996 (34,674,000)

APH=Antepartum haemorrhage; PPH=Postpartum haemorrhage

Of injuries-related deaths, approximately half (10.7%) were due to suicide. Of 425 women who were pregnant when they died due to injuries, 201 (47.4%) were due to suicide, 65 (15.3%) due to homicide, and 75 (17.6%) due to unintentional injuries, and for 84 (19.8%), the intent was unknown.

Women who died due to obstetric causes, pregnancy-related medical causes, or from abortion were more likely to be aged 20–29 or 30–39 years (Table 3). Women who died due to medical causes were most commonly aged 40–50 years. The mean age at death was lowest among women who died due to injuries. The vast majority (71.2%) of deaths due to injuries occurred among women aged 10–29 years. Of all the reported cases, a large proportion (45.2%) of the women were illiterate. Of all the reported deaths, 76.6% occurred among married women. The proportion of women dying from abortion-related causes who were in the middle/upper middle economic status was markedly lower (3.8%) than the proportion among women dying from other causes (range 18.5–27.4%). The percentage of women who were not married was highest among women dying from injuries (24.3%) or medical causes (15.1%). Overall, 86.8% of women were village residents. The percentage of women who were village residents was actually higher (more than 95%) among deaths from medical and injury causes during pregnancy.

**Table 3:** Selected characteristics of women aged 10–50 years reported to have died, Bangladesh, 1996–1997, by cause of death

Characteristics	Causes of death						
	Pregnancy			Medical		Injury	
	Total (n= 28,998)∗	Obstetric cause (n= 7,086)	Abortion-associated cause (n=1,476)	Pregnancy associated cause (n=259)	Other medical causes (n= 13,502)	Pregnancy-associated cause (n=425)	Other injuries (n= 6,186)
Age (years)							
10–19	17.7	12.6	8.2	10.1	14.5	25.2	32.7
20–29	33.6	50.4	36.1	43.3	21.6	55.2	37.9
30–39	27.8	30.3	43.9	37.9	29.1	16.5	18.7
40–50	19.0	4.2	7.9	7.7	33.5	1.2	9.1
Unknown	1.9	2.5	3.8	1.1	1.4	1.9	1.7
Mean age	29.1	26.6	29.4	28.5	32.7	23.7	24.6
(SD)	(10.1)	(6.6)	(6.9)	(7.2)	(11.2)	(5.7)	(9.1)
Education							
Illiterate	45.2	43.1	52.5	58.0	47.6	50.7	40.0
Incomplete Primary	15.3	15.5	14.3	20.9	14.4	22.2	17.0
Incomplete Secondary	9.4	8.1	8.4	9.2	7.4	14.9	14.9
SSC/HSC/higher	2.2	2.3	1.4	1.6	1.5	4.2	3.7
Unknown	27.9	30.9	23.4	10.4	29.1	8.0	24.4
Economical status							
Poor	33.5	33.2	43.0	47.1	32.4	44.6	32.9
Lower middle	21.0	19.8	31.7	23.9	20.0	25.7	21.9
Middle/upper middle	19.0	18.5	3.8	21.2	19.1	27.4	22.6
Unknown	26.4	28.4	21.5	7.7	28.6	2.4	22.6
Marital status							
Presently married	76.5	90.9	85.8	97.3	73.1	89.6	64.3
Presently not married	12.8	0.4	5.7	1.2	15.1	9.9	24.3
Unknown	10.7	8.7	8.5	1.5	11.9	0.5	11.4
No. of living children							
0	9.7	19.4	5.0	16.2	3.5	26.7	11.7
1–2	20.5	24.9	18.9	33.2	16.5	36.8	23.4
3–4	18.0	17.2	28.3	22.4	20.3	13.0	11.9
5+	10.9	8.5	16.0	16.2	14.8	3.5	4.5
Unknown	40.9	30.0	31.8	12.0	45.0	20.0	48.6
Mean no. of children	2.7	2.1	3.2	2.5	3.3	1.5	2.0
(SD)	(2.0)	(1.9)	(1.9)	(2.1)	(2.0)	(1.5)	(1.7)
Residence							
Division/district sadar	5.4	5.0	3.9	0.8	3.8	0.7	6.6
Upazila sadar	4.0	4.6	2.1	3.1	6.2	1.4	3.1
Village	86.8	86.3	90.7	95.4	85.2	97.4	89.2
Unknown	4.1	4.2	3.4	0.8	4.8	0.5	3.1

∗ Includes 65 cases with unspecified cause not shown separately in this table; HSC=Higher secondary certificate; SD=Standard deviation; SSC=Secondary school certificate

The percentage of deaths from different causes varied by age-group (Table 4). Of women aged 10–19 years, the most common causes of death were injuries (39.3%) and other medical causes (38.0%). Of those aged 20–29 years, the most common causes of death were pregnancy-associated causes (42.2%), other medical causes (30.0%), and injuries (24.1%). Of those aged 30–39 years, the most common causes of death were other medical causes (48.7%) and pregnancy-associated causes (34.7%), while for those aged 40–50 years, the most common causes of death were other medical causes (81.8%).

**Table 4:** Reported causes of deaths among women aged 10–50 years, by age-group, Bangladesh, 1996–1997

Cause of death	Age-group (years)			
	10–19 (n=5,140) %	20–29 (n=9,738) %	30–39 (n=8,054) %	40–50 (n=5,522) %
Pregnancy cause	19.7	42.2	34.7	7.1
Direct obstetric cause	15.1	30.5	21.1	4.1
Eclampsia	9.2	13.9	6.3	0.8
Obstructed labour	1.8	3.7	3.5	0.8
PPH/APH	1.9	6.0	5.4	0.8
Retained placenta	0.9	3.0	3.0	0.7
Tetanus	0.4	1.1	0.7	0.2
Other obstructed causes	0.9	2.8	2.3	0.8
Indirect obstetric cause	2.2	6.2	5.6	1.3
Abortion-related cause	2.4	5.5	8.0	2.1
Induced	2.2	5.1	7.8	2.1
Spontaneous	0.1	0.3	0.2	0.0
Pregnancy-associated medical cause	0.5	1.2	1.2	0.4
Other medical causes	38.0	30.0	48.7	81.8
Infectious diseases	14.9	8.8	11.4	12.7
System disorders	7.5	5.5	8.7	17.0
Cardiac problems	5.5	5.9	11.6	28.0
Anaemia/malnutrition	3.1	3.4	5.7	7.4
Hepatobiliary conditions	3.9	4.1	5.0	5.1
Cancer	3.0	2.2	6.2	11.6
Pregnancy-associated injury	2.1	2.4	0.9	0.1
Other injuries	39.3	24.1	14.3	10.2
Suicide	21.7	13.2	6.3	2.9
Homicide	1.1	1.4	0.8	0.5
Accidental	6.4	2.3	3.0	3.7
Unknown intent	10.2	7.2	4.2	3.1
Unspecified cause	0.4	0.3	0.2	0.1
All causes	100.0	100.0	100.0	100.0

∗ Women population in 1,000s; APH=Antepartum haemorrhage; PPH=Postpartum haemorrhage

The percentage of deaths from different causes also varied by administrative division (Table 5). Other medical causes made up the largest percentage of deaths in all divisions, except Khulna where injury constituted the highest percentage. The largest proportion of deaths from other medical causes was in Barisal (57.0%). The proportion of deaths that were due to cardiac and systemic disorders was highest in Barisal (24.4%) and lowest in Khulna (15.9%). The percentage of deaths from infectious diseases was highest in Sylhet (16.8%) and lowest in Khulna (5.7%). The percentage of deaths from pregnancy-associated causes, including direct obstetric causes, was highest in Sylhet (38.9% and 27.0% of all deaths respectively). Obstetric deaths in Sylhet were mostly due to eclampsia and haemorrhage (11.8% and 6.4% of all deaths respectively).

**Table 5:** Reported causes of death among women, aged 10–50 years, by division, Bangladesh, 1996–1997

Cause of death	Dhaka (n=7,328) %	Chittagong (n=5,123) %	Rajshahi (n=7,979) %	Khulna (n=4,552) %	Barisal (n=2,277) %	Sylhet (n=1,739) %
Pregnancy cause	30.6	33.3	27.4	23.1	30.2	38.9
Direct obstetric	21.4	22.4	18.0	15.3	22.2	27.0
Indirect obstetric	5.1	5.4	3.7	2.6	3.1	6.7
Abortion-related	4.1	5.5	5.7	5.2	4.9	5.2
Pregnancy-associated medical cause	1.0	1.3	0.9	0.6	0.6	0.2
Medical cause	50.4	45.1	47.3	34.9	57.0	48.1
Pregnancy-associated injury	0.9	2.1	1.6	1.9	0.7	1.3
Other injuries	16.5	18.2	22.7	39.5	11.6	10.5
Unspecified cause	0.7	0.0	0.2	0.0	0.0	0.0
All causes	100.0	100.0	100.0	100.0	100.0	100.0

Of all cases of non-abortion deaths, 24.3% did not receive any medical treatment prior to death; this group included 0.1% of women who died on the way to obtaining medical treatment (Table 6). Approximately, half of the women received medical treatment either from a registered physician or at a clinic or a hospital (47.8%). A large proportion (25.6%) of cases received treatment from other sources, including unregistered (village) doctors, homeopathic practitioners, kabiraj, pharmacists, traditional birth attendants, and religious healers. Women who died from pregnancy-associated injury and other injury-related causes were most likely not to have received treatment prior to death (62.4% and 51.3% respectively), whereas women who died due to medical causes were least likely not to have received treatment (13.3%). Women who died due to medical and obstetric causes were most likely to have received treatment from a registered physician or a hospital or a clinic (54.3% and 51.1% respectively).

**Table 6:** Medical treatment received prior to deaths among women aged 10–50 years, by cause of death∗, Bangladesh, 1996–1997

Medical treatment prior to death	Obstetric-related deaths (%)†	Medical-related deaths (%)†	Pregnancy-associated medical-related deaths (%)†	Pregnancy-associated injury-related deaths (%)†	Other Injury-related deaths (%)†	Total (%)‡
No treatment received	19.5	13.3	18.9	62.4	51.3	24.3
Admitted to a hospital/clinic or treated by a registered doctor	51.1	54.3	42.1	15.3	32.8	47.8
Treated by a nurse, FWV, FWA, or MA	11.1	7.9	6.9	2.4	7.5	8.5
Other¶	24.9	32.3	37.1	15.8	12.3	25.6
Do not know/unknown	5.7	7.5	5.8	7.1	4.3	6.4

∗ Medical treatment prior to death is not presented for deaths from abortion as this question was not asked for these cases;

† Column percentage may add to more than 100 as more than one type of treatment prior to death was reported for some cases;

‡ Includes 65 cases of unspecified deaths not shown in a separate column;

¶ Includes treatment by unregistered (village) doctors, homeopathic practitioners, kabiraj, pharmacists, compounders, trained traditional birth attendants, traditional birth attendant, religious healers, aya, neighbour, or relative

FWA=Family Welfare Assistant; FWV=Family Welfare Visitor; MA=Medical Assistant

## DISCUSSION

This study was modelled after the study conducted in the 1978–1979 national survey of health functionaries in hospitals and non-hospital facilities on pregnancy-associated deaths, which covered 795 health facilities to enable the assessment of pregnancy-associated deaths. Compared to local population-based mortality rates, the 1978–1979 study identified an estimated 7.5% of pregnancy-associated deaths (2). Application of the 28,998 deaths identified in our study to a population estimate of women aged 10–49 years in Bangladesh in 1996 resulted in an unadjusted mortality rate of 83.6 deaths per 100,000 women. A small local study, conducted in Matlab subdistrict, estimated that the annual mortality rate among women aged 15–44 years, during 1976–1985, was 290 per 100,000 women (7). The researchers had used information from the control areas of the Matlab Demographic Surveillance System. This implies that we identified about 28.8% (83.6/290) of deaths among women.

Several factors probably contributed to this study not being able to identify a larger proportion of deaths. The study was not conducted in four of the 64 districts of Bangladesh. In districts where the study was conducted, we did not collect information from all the government facilities, such as district hospitals, Upazila Health Complexes, and Union Health and Family Welfare Centres, that also provide services to women. Classification of causes of death from hospital records might have been affected by its documentation in the death certificate. Sometimes, the listed causes of death was cardio-pulmonary failure and not the specific disease. In some cases, the case history and treatment sheet helped in assessing the causes of death. We do not know the extent of errors relating to the causes of death in medical records in Bangladesh, but our categories of death are broad and may be less likely to have errors in assessing the causes of death.

Our study primarily aimed at covering the public facilities in Bangladesh. The under-representation of private facilities, which presumably provide better healthcare to healthier populations, may bias the findings of this study to reflect disproportionately greater health problems than the overall population has. We do not see this as a disadvantage as most studies are biased the other way. Around the time of this study, private facilities accounted for only about 28% of hospital beds in the country (12). Although the FWAs were the major source of case reports, we were only able to interview approximately 60% of FWAs in Bangladesh (12).

Another limitation of the study is that we could not validate the reported cases of deaths among women. However, deaths are major events, and people who knew the deceased may be likely to remember the circumstances of the death (7). In countries, such as Bangladesh, where the vast majority of deaths occur at home, medical records are often unavailable, and family members are reluctant to talk about circumstances of the death. Thus, collecting information from knowledgeable people within the community may be an effective way for identifying and classifying such deaths (2,7,13). In addition, the World Health Organization recommends the use of verbal autopsies (e.g. medical classification of information reported by non-medical persons) to determine the causes of death in countries, such as Bangladesh (14). But verbal autopsies based on knowledge of health-facility workers or medical record information may be substantially more limited and more subject to error than verbal autopsies based on interviews with family members. We could not independently assess the quality of information respondents or medical records provided. On the positive side, the patterns of causes of death did not vary greatly between the data from the medical facilities and the data from the FWAs.

A large number of deaths in our study were reported by the health functionaries (FWAs) who are supposed to visit households in their areas. The fact that sociodemographic information was available for most cases suggests that respondents usually had knowledge about the deceased woman. There were also a large number of cases where sociodemographic information was not available. As the FWAs have some medical knowledge, the information provided may be more likely to be medically correct. These health workers may have the capacity to recognize and distinguish the major categories of causes of death that we used in this study, but we lacked independent verification of this.

A major strength of our study is that it was conducted nationally with medical chart review and/or informant interviews at more than 4,700 health facilities throughout the country and collected reports on nearly 29,000 deaths. The large sample size increased the ability to determine deaths from different causes among women aged 10–50 years in Bangladesh. In addition, the cost of conducting the study was low (approximately $100,000) and, therefore, this may be an additional factor for consideration of this type of study.

We hope that the findings of this study will lead to more focus on all causes of deaths among women aged 10–50 years in Bangladesh. The regional differences in the percentage of deaths from various causes probably indicate the need to develop the prevention and treatment strategies that reflect on such differences and for local-level planning and implementation. In addition, considering socioeconomic and age-group differences can possibly make disease-prevention strategies to be more effective.

The number of obstetric deaths can be effectively reduced through improving the availability and use of prenatal and obstetric care. All pregnant women should receive appropriate prenatal care, and strengthening the availability of these services locally can help achieve this. Facility-based deliveries are needed for high-risk pregnancies, and deliveries at home should be conducted by adequately-trained persons with appropriate referral of complications. Strengthening emergency obstetric care can improve the availability of adequate services to reduce the number of deaths from obstetric complications. The number of deaths due to tetanus could be reduced by providing tetanus toxoid (TT) to women during pregnancy and possibly even before they become pregnant (8). Among various prevention strategy considerations, providing tetanus toxoid before reproductive age can prevent tetanus among women who may not have received prenatal care (15).

Further support of contraceptive-use can reduce deaths due to the number of abortions through prevention of unintended pregnancies. Appropriate post-abortion care can reduce the number of deaths due to abortions. Those having abortions need to access appropriate medical services immediately. Easy access to appropriate medical treatment is also needed. These medical services are especially needed for women who are poor or who live in rural areas.

Infectious diseases are a common problem in Bangladesh and in other developing countries. Effective and community-oriented strategies need to be implemented that can reduce the number of deaths from infectious diseases. Interventions and programmes to address infection diseases need to be considered by undertaking prevention measures that can reduce infections and increasing the availability and use of appropriate medical treatment.

Potential strategies for improving overall health of women are important. These strategies include focus on primary prevention of chronic diseases, such as cancer and cardiovascular diseases. These diseases are very important causes of death among women aged 10–50 years in Bangladesh. Strategies to achieve better health may include increasing awareness about chronic diseases as a major health problem among women in Bangladesh. Increased awareness and information can possibly lead to more focus on chronic disease-related problems. Better knowledge about chronic diseases may increase the ability of women to improve health. Specific strategies are needed that could help improve health knowledge among women.

Our results indicate that a substantial number of deaths occurred due to intentional and unintentional injuries. Approximately 21.3% of deaths resulted from injuries, and these were commonly suicide-associated deaths. Injury-related deaths also varied by region. Although it is possible that this variation is reporting differences between regions, these may also be real. Strategies to prevent deaths due to injuries could include raising awareness about injuries. Programmes for promoting safer practices through public education may also be a helpful prevention strategy. Further studies are needed about to understand the reasons why the variations occur.

A concerning finding of this study is that about 24% of women who died did not receive any medical treatment, and only about 48% received treatment from a registered physician, a hospital, or a clinic before death. Possible reasons for not having received medical treatment may include the lack of time or lack of opportunity to reach a physician or a hospital, lack of availability and other barriers, such as financial or transport-related barriers, to accessing adequate medical services, and lack of knowledge and awareness of individuals about accessing appropriate medical treatment (7,16,17).

The need for more efforts and strategies for the prevention of diseases in Bangladesh have been previously reported (18). Strengthening of local-level services through health facilities and outreach health functionaries are needed. These strategies can help prevent deaths among women in Bangladesh.
